# Factors affecting the adoption of green prevention and control techniques by family farms: Evidence from Henan province of China

**DOI:** 10.3389/fpsyg.2022.1015802

**Published:** 2022-12-22

**Authors:** Tingting Chen, Xiaojing Lu, Zhanyong Wu

**Affiliations:** ^1^School of Economics and Trade, Henan Polytechnic Institute, Nanyang, China; ^2^Economics and Management school, Wuhan University, Wuhan, China; ^3^Fanli Business School, Nanyang Institute of Technology, Nanyang, China

**Keywords:** green prevention and control techniques, adoption behavior, IMB model, vegetable farmers, ordered probit model

## Abstract

Encouraging farmers to adopt green prevention and control techniques (GCTs) is conducive to ensuring the quality and safety of agricultural products, the ecological environment and agricultural production in China. To explore the factors influencing vegetable farmers’ adoption of GCTs, this study utilized the “information-motivation-behavior” intervention (IMB) model and ordered logistic model to empirically study the adoption behavior of 653 vegetable farmers in Henan Province, China. Estimation results indicated that the IMB model significantly predicted farmers’ adoption of GCTs: 1) From the perspective of adoption decisions, 88.82% of the farmers have adopted GCTs, but the degree of adoption is low. 2) the farmers’ adoption of GCTs IMB model specifies that higher levels of GCTs information, motivation, and behavioral skills should result in a greater likelihood of engaging in GCTs adoption behavior. 3) Motivation and behavioral skills are activated through information. 4) Finally, motivation can indirectly affect farmers’ GCTs adoption behavior through behavioral skills. The results of this study support the need for the government to promote the use of GCTs for vegetable pest control, as well as advance integrated prevention and control in the agricultural industry.

## 1. Introduction

Currently, irregularities such as increased chemical pesticide application, accelerated application frequency and shorter intervals are common among Chinese farmers, and excessive chemical pesticide application has caused increased agricultural surface pollution and agroecosystem degradation and it poses a serious threat to the quality and safety of water bodies and soil (Lou et al., 2021; Yu et al., 2021a). To reduce and control the usage of chemical pesticides, and provide ecological and environmental safety, thereby promoting the sustainable development of agriculture, China’s government is committed to promoting the Chinese practice of green prevention and control techniques (GCTs). GCTs is the Chinese concept of integrated pest management (IPM) and prioritizes adopting resource-saving and environmentally friendly technical measures such as ecological regulation, biological control, physical control, and scientific pesticide use. Furthermore, it helps reduce crop losses, increase yields, and improve the net income and welfare of farmers (Wang et al., 2015; Gao et al., 2017). However, compared to traditional production technology methods, GCTs require higher labor inputs and more stringent operational requirements. Farmers, as “rational economic people,” will inevitably consider factors such as costs, benefits, and risks, which lead to differences in the adoption of GCTs by farmers and make it difficult to achieve large-area diffusion of green control technologies (Gao et al., 2017; Gao and Niu, 2019). At present, the use of GCTs in China remains mainly experimental and implemented at a small scale, and has become one of the “bottlenecks” limiting the sustainable development of Chinese agriculture (Yu et al., 2020a, 2021a). Demand from farmers, who constitute the micro decision-making body of agricultural production and operation, would be the basis for the successful application of GCTs. Therefore, we explored the influencing factors of Chinese farmers’ adoption of GCTs.

Scholars have conducted extensive and in-depth studies on the factors that influence the adoption behavior of GCTs by farmers. It is generally believed that GCTs adoption is related to farmers’ individual characteristics (Abebaw and Haile, 2013; Murage et al., 2015; Kolleh, 2016; Luo et al., 2022), resource endowment characteristics (Allahyari et al., 2016; Gao et al., 2019a; Luo et al., 2019; Niu et al., 2022), and cognitive characteristics (Kabir et al., 2017; Gao et al., 2019b). Moreover, GCTs adoption is also related to external factors such as government regulation (Gao et al., 2019c), technology integration (Benelli et al., 2017; Fatima et al., 2022), and technical training (Geng et al., 2017). By combing the literature, the current domestic and international studies on the factors influencing farmers’ GCTs adoption behavior mainly show the following characteristics. First, the studies are mostly about farmers’ own resource endowment (Xiong and He, 2020), social networks (Gao et al., 2019a), economic incentives (Geng et al., 2017) and technical characteristics (Gao et al., 2020; Liu et al., 2022) effects on farmers’ GCTs adoption behavior, and there is a lack of research on farmers’ psychological motivation. Second, the research objects are mostly about whether farmers adopt GCTs, ignoring the gradual process of GCTs adoption and the degree of adoption by farmers (Lin et al., 2021). Third, the research subjects are mostly food crop farmers, ignoring the research on farmers in other farming industries (Korir et al., 2015; Allahyari et al., 2016; Geng et al., 2017). Agricultural non-point source pollution is hidden, scattered, and difficult to find. On the premise of not changing the individual’s awareness and concept of green prevention and control, there are no prescribed best practices for implementing effective behavioral interventions targeting constraining farmers to adopt GCTs. Psychologically-based interventions may be useful in explaining and ultimately promoting the adoption behavior of GCTs by farmers. As such, the current study incorporates the information-motivation-behavior intervention (IMB) model into the theoretical analysis framework to explore the influence of psychological factors on farmers’ adoption of GCTs.

The IMB model was first designed by Fisher and Fisher (1992), who took behavioral intervention as the starting point for determining behavioral change and divided the factors affecting individual behavioral change into three parts: information, motivation, and behavioral skill. Among them, information refers to the knowledge related to individual behavior transmission that can lead individuals to think and transform their behavior. Motivation refers to the individual’s attitude (personal motivation) and subjective norms (social motivation), including the individual’s expectations of the costs and benefits of adopting the behavior, as well as social norms. Behavioral skills refer to the skills that facilitate the effective implementation of behavior, which provide a guaranteed basis for behavioral safety (Fisher et al., 2014). The above three can act as mutually independent entities to influence individual behavior directly, or they can interact with each other to influence individual behavior indirectly. The IMB model further distinguishes itself from other behavior change models (e.g., the theory of reasoned action, Fishbein and Ajzen, 1975; transtheoretical model, Prochaska et al., 1992) by outlining a three-step approach to designing interventions that are tailored to a specific population: elicitation, design and implementation, and evaluation. Thus, the IMB model provides a theoretical understanding of behavior change and a guide for designing theory-informed interventions. The IMB model has been used successfully in multiple health domains (Fisher et al., 2014) and is increasingly used in other fields, including recycling behavior (Seacat and Northrup, 2010), farmers’ willingness to adopt agricultural technology (Zhang et al., 2020), and water conservation behavior (Ehret et al., 2021). Based on this, this paper attempts to incorporate the “information-motivation-behavior skill” behavioral intervention model into the theoretical framework to analyze farmers’ GCTs adoption behavior and provide new ideas for exploring effective paths to enhance farmers’ GCTs adoption behavior.

## 2. Theoretical framework and research hypothesis

### 2.1. Information and farmers’ GCTs adoption behavior

According to the IMB model, information relevant to performing the desired behavior is a prerequisite to the correct and consistent performance of that behavior (Fisher et al., 2006). Within the domain of green agricultural production, researchers have demonstrated the important role that information has in promoting and maintaining farmers’ green production behavior (Korir et al., 2015 and Zhang et al., 2020). Persuasion theory believes that by disseminating a certain aspect of information, individuals will deepen their understanding of this aspect of knowledge, and when they have enough of this information, they will often persuade themselves to actively participate, and then affect the individual’s related willingness and decision-making (Lou et al., 2021; Luo et al., 2022). In the incomplete information context, information intervention in the information channel mainly shapes farmers’ behavior through the paths of sharing information resources, enhancing risk prevention and control, and reducing transaction costs, and farmers with more information channels also receive stronger information intervention and have a greater likelihood of adopting green prevention and control technology behavior (Zhang et al., 2020; Yu et al., 2021a; Shi and Zhang, 2022). In addition, farmers also have information needs in the process of green prevention and control technology adoption decisions, and whether the information needs of farmers can be met mainly depends on whether farmers have rich information access channels (Yan and Liu, 2022). Farmers with rich information channels can obtain more positive information in the process of information transfer and can give full play to the value of such information, and the advantages of farmers’ adoption of green prevention and control technology will be more obvious (Wang et al., 2021a; Li et al., 2022). Based on this, the following hypothesis is proposed in the paper.

*H1*. Information acquisition has a significant positive effect on farmers' GCTs adoption behavior.

### 2.2. Motivation and farmers’ GCTs adoption behavior

The IMB model posits that behavioral change is more likely when individuals are motivated (Fisher et al., 2014). We define motivation as the driving force that motivates individual behavior, and gaining more profit, appreciation, and avoiding punishment are the most fundamental motives that promote farmers’ green prevention and control technology behavior. According to the IMB model, motivation exists on two levels: individual and social with both forms of motivation being influenced by differing sources (Seacat and Northrup, 2010). On the personal level, a high level of motivation and positive attitude toward performing a behavior is based upon the belief that one can successfully engage in the desired behavior and that the outcome of engaging in this behavior will be beneficial to the self. Further, individuals need to believe that the costs associated with performing the desired behavior outweigh the costs of engaging in that behavior (Ehret et al., 2021). At the social level, motivation is based on individuals’ perceptions of social support (e.g., government or cooperatives) for engaging in the desired behavior (Gao et al., 2019a). With regard to green agricultural production behavior, it is well-recognized that personal attitudes and social support are critical to engaging in pro-environmental behavior (Gao et al., 2017). In the process of agricultural production, farmers, as “rational economic people,” will always make the best production decision. On the one hand, farmers have the most fundamental production motivation to pursue maximum economic benefits, thus, the higher the market value recognition degree of green agricultural products produced by farmers, the greater the possibility of farmers adopting green prevention and control technology (Geng et al., 2017). On the other hand, consistent with the IMB Model, the arrangement of relevant government incentives and restraint policies will guide and standardize farmers’ production behavior (Lin et al., 2021). Agricultural technology subsidies as a form of financial compensation are the most effective external incentive for behavioral interventions and can promote farmers’ active participation in rural public environmental governance (Gao et al., 2019b; Lou et al., 2021). However, to avoid the risk of penalties associated with non-environmental behaviors, farmers will continue to adopt environmentally friendly production technology behaviors (Mao et al., 2021; Hu et al., 2022). Based on this, the following hypothesis is proposed.

*H2.* Motivation has a significant positive effect on farmers' GCTs adoption behavior.

### 2.3. Behavioral skills and farmers’ GCTs adoption behavior

The final critical prerequisite of engaging in the desired behavior is the possession of the appropriate behavioral skills by individuals, to successfully perform appropriate behaviors (Fisher et al., 2014). Behavioral skills consist of individuals’ objective abilities as well as their perceived self-efficacy concerning the performance of appropriate behaviors (Seacat and Northrup, 2010). Green prevention and control technology adoption behavior skill refers to the green prevention and control technology that farmers understand and master (Gao et al., 2019c; Luo et al., 2022). For most farmers, green prevention and control technologies have technical barriers, such as difficulty in operation and high requirements, which are the key reasons that currently inhibit farmers’ adoption of green prevention and control technologies (Shi and Zhang, 2022; Yu et al., 2022). However, participation in agricultural technology training can help farmers effectively master agricultural technology skills, deepen their understanding of agriculture-related technologies and policies, and improve the accessibility of agricultural technologies to farmers, thus breaking down agricultural technology barriers (Gao et al., 2019c; Zhang et al., 2020). At the same time, agricultural technology promotion and training can effectively reduce the use of chemical inputs in agricultural production, improve farmers’ knowledge and understanding of agricultural green production technologies in the process of technical guidance, and promote the rationalization of farmers’ chemical input behavior (Gao et al., 2017; Niu et al., 2022). Consistent with the IMB model, higher levels of perceived GCTs behavioral skills among individuals should lead to a greater likelihood that these individuals will engage in green prevention and control technology adoption behavior. Based on this, the following hypothesis is proposed.

*H3.* Behavioral skills have a significant positive effect on farmers' GCTs adoption behavior.

### 2.4. IMB model and farmers’ GCTs adoption behavior

Although information and motivation may have direct effects on behavior when the target action does not require complex skills (Fisher et al., 2014), the IMB model proposes that information and motivation often indirectly influence actions *via* behavioral skills, consistent with the reasoned action model (Fishbein and Ajzen, 1975). That is, higher levels of information and motivation lead individuals to believe they can engage in and/or objectively be able to engage in the target behavior, moving them to acquire the skills necessary to undertake the behavior (Seacat and Northrup, 2010; Ehret et al., 2021). The transmission of intergroup information can make farmers feel the correct social norms and standards, enhance their motivation for behavior change (Zhang et al., 2020; Zhao et al., 2021), and provide new knowledge and techniques that are acceptable to farmers, ultimately creating an atmosphere conducive to their behavior change (Qin and Lv, 2020; Wu and Zhou, 2021; Shi and Zhang, 2022). In addition, by breaking through the barrier of information blockage through multiple channels of positive information, farmers not only help break the technical barrier of high cost and difficulty in mastering but also help reduce their risk perception of adopting new technology behavior (Zhou et al., 2022), improve their perception of economic benefits, and promote positive behavioral motivation, which in turn drives farmers’ enthusiasm to adopt ecological farming behavior (Mao et al., 2021; Wang et al., 2021b). Moreover, farmers with higher perceptions of economic benefits and risk preferences are more motivated to engage in agricultural production, are happy to participate in new technology training activities and are more inclined to try new technologies with high returns and risks (Wang et al., 2015; Yu et al., 2020b, 2021b; Hu et al., 2022). Based on this, the following hypotheses are proposed.

*H4.* Information acquisition has a significant positive effect on farmers' GCTs adoption motivation.

*H5.* Information acquisition can act on farmers' GCTs adoption behavior through motivation mediation.

*H6.* Information acquisition has a significant positive effect on farmers' GCTs behavioral skills.

*H7*. Information acquisition can act on farmers' GCTs adoption behavior through behavioral skills mediation.

*H8.* Motivation has a significant positive effect on farmers' GCTs behavioral skills.

*H9.* Motivation can act on farmers' GCTs adoption behavior through behavioral skills mediation.

**Figure 1:**
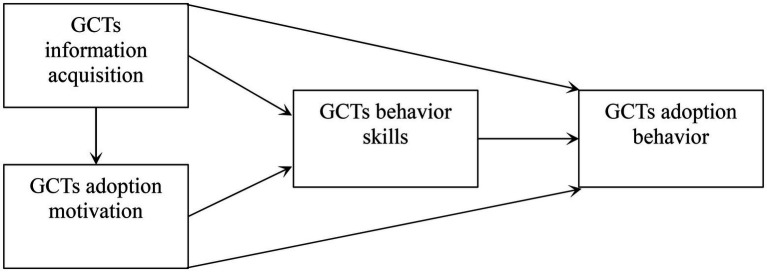
The theoretical framework for examining farmers’ adoption of GCTs in IMB model.

In summary, the theoretical framework for examining the applicability of the IMB model in predicting farmers’ adoption of GCTs is shown in Figure 1. To test these hypotheses, we carry out a survey that is presented in the next part of this paper.

## 3. Research design and methodology

### 3.1. Questionnaire design

We designed a questionnaire related to the behavior of vegetable farmers adopting GCTs by referring to the scale designed by previous studies on the different research objects’ behavior of green production technology. Our questionnaire includes five parts.

Part 1 was vegetable farmers’ socio-demographic characteristics, production and operation characteristics. *Householder characteristics*. (1) Gender. We code the gender variable such that “male” equals 1 and “female” equals 0. (2) Age. We measure a householder’s age as his/her actual age. (3) Degree of education. We use a householder’s number of years of education to measure the education variable. (4) Village cadre status. Village cadre members are usually rural elites who have more information about green production conditions and new technologies and are likely to sign long-term written contracts and adopt new technologies in response to the government’s call to lead by example (Jiang et al., 2018; Zeng et al., 2019). We code this variable such that “yes” equals 1 and “no” equals 0. *Production and operation characteristics*. (1) Incentives for green planting. Referring to Shi and Zhang (2022)’s method of dealing with the problem, we code this variable such that “yes” equals 1 and “no” equals 0. (2) Number of laborers. We measure the labor force quantity as the number of household laborers and long-term employees. (3) Degree of participation in agricultural orders. Referring to Shi and Zhang (2022)’s method of dealing with the problem, we measure degree of participation in agricultural orders on a 5-point Likert scale.

Part 2 investigated vegetable farmers’ adoption behavior of green prevention and control techniques. Based on the “one control, two reduction and three basic” surface pollution prevention and control objectives proposed by the Ministry of Agriculture in 2020 and taking into account the actual situation in Henan Province, five specific indicators were selected to measure the adoption of GCTs by farmers: pollution-free pesticide application, organic fertilizer application, green fertilizer planting, soil formula fertilizer application and mulch disposal. The farmers who participated in 0 items were never adopted and assigned a value of 1; those who participated in 1–2 items were occasionally adopted and assigned a value of 2; those who participated in 3 items were frequently adopted and assigned a value of 3; those who participated in 4 items were often adopted and assigned a value of 4; and those who participated in 5 items were always adopted and assigned a value of 5. The higher the score is, the higher the degree of adoption of GCTs by farmers.

Part 3 is GCTs information acquisition. Narrow information channels are a serious obstacle to farmers’ access to information resources and an important factor affecting the degree of farmers’ GCTs adoption behavior (Yang et al., 2020). Therefore, “information access channels: government, cooperative, neighborhood or friends, media” was selected as the measure of information, with the following criteria: 0 means no information channels, 1 means 1 information channel, 2 means 2 information channels, and 3 means 3 information channels, 4 means 4 information channels.

Part 4 is GCTs adoption motivation. The most fundamental motivation of smallholder farmers is to pursue profit maximization, and behavioral costs, rewards and penalties are the key factors affecting smallholder motivation (Shi and Zhang, 2022). Drawing on the following scholars’ research (Zhang et al., 2020; Xiong et al., 2021), we selected the following six specific indicators as the measure of motivation: “GCTs adoption behavior is conducive to improving the quality of vegetables,” “GCTs adoption behavior is conducive to obtaining green certification,” “GCTs adoption behavior is conducive to increasing planting income,” “GCTs adoption behavior is beneficial to save production cost,” “government subsidies for GCTs promotion services” and “local penalties for agricultural surface source pollution.”

Part 5 is GCTs behavior skills. Agricultural technology promotion and training are important ways to enhance farmers’ knowledge of agricultural technology. Based on the method of Shi and Zhang (2022) and Ehret et al. (2021), we selected “whether to participate in various types of training such as agricultural land protection technology,” “whether to often communicate with agricultural technicians,” “whether to often learn green prevention and control technology publicity materials” to measure GCTs behavioral skills.

### 3.2. Data sources

#### 3.2.1. Introduction to the study area

**Figure 2:**
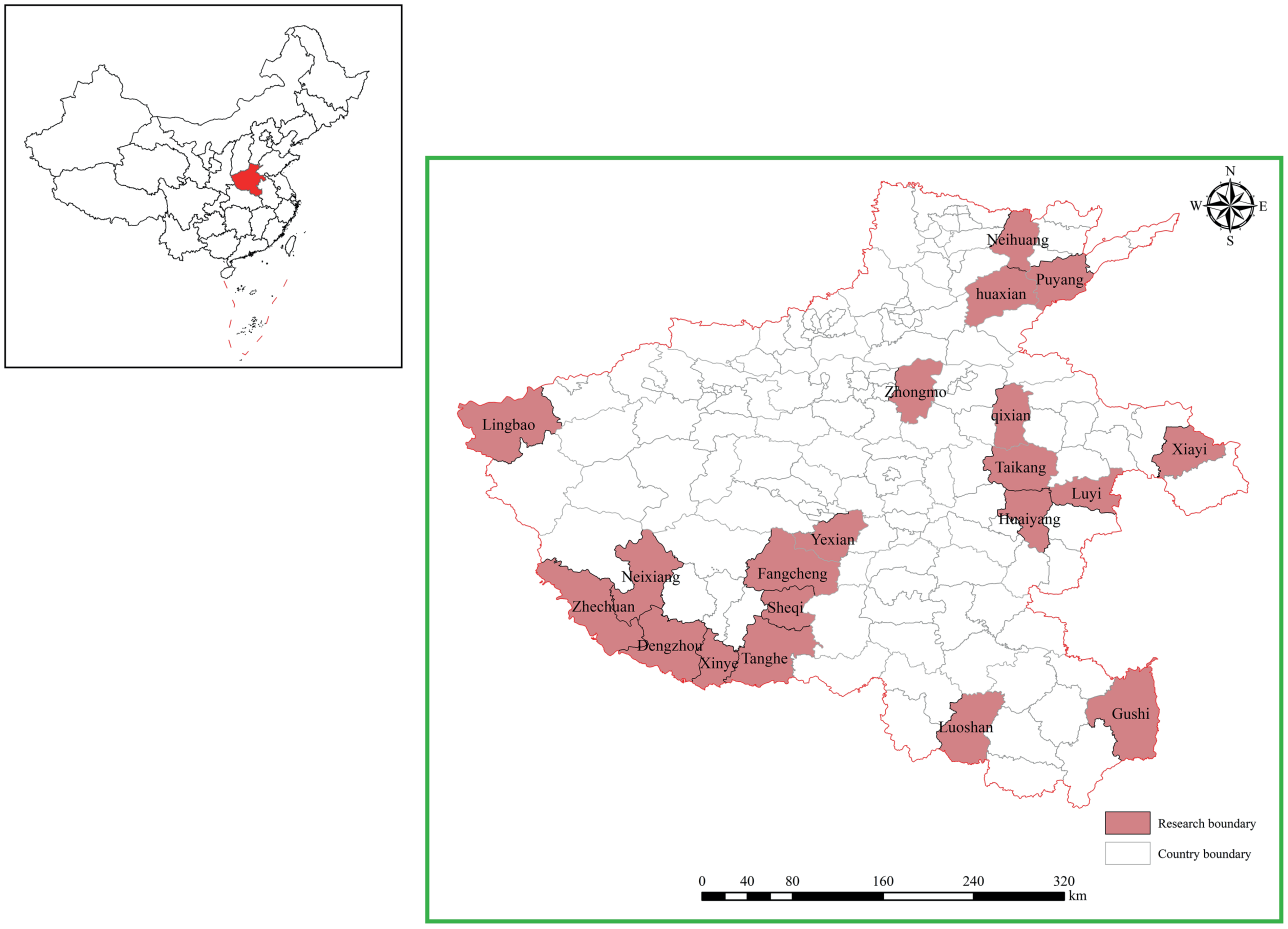
The position of Henan province in China and study area.

The data for this study were obtained from field research conducted by the research team from April to October 2021 on vegetable farmers in Henan Province (as shown in Figure 2). The sample selection area is based on the following considerations. On the one hand, Henan Province, a big vegetable province in China, has an average vegetable planting area of about 1.88 million hm^2^ every year, and its total output ranks among the top in China. Henan Province is rich in vegetable varieties, with more than 150 kinds of vegetables in more than 10 categories. It is an important producing area of high-quality Chinese cabbage, tomato, radish, pepper, ginger, garlic and other crops, providing a large number of vegetables for domestic and foreign regions every year. Therefore, taking Henan Province as the investigation area is helpful to obtain a comprehensive and sufficient sample of vegetable farmers. On the other hand, due to the unsustainable agricultural production modes such as intensive use of chemicals, over-utilization of cultivated land and over-exploitation of groundwater in some areas in the early stage, the soil organic matter in Henan Province is only 19.2 g/kg in 2020, which is lower than the national average level of 24.9 g/kg of cultivated land. As the “hardest hit area” where pesticides and fertilizers are intensively used, the soil quality of vegetable fields is even less optimistic. Facing the urgent situation of agricultural green production transformation. In recent years, Henan provincial government has adhered to the plant protection policy of “putting prevention first and comprehensive prevention and control,” mainly through five core technical means: ecological regulation, agricultural control, physical and chemical inducement control, biological control and scientific drug use, and strive to achieve a green prevention and control coverage rate of 55% by 2025. Therefore, taking vegetable farmers in Henan Province as the research object has good representativeness, data support and practical significance.

#### 3.2.2. Sample selection and data collection

The sample is vegetable farmers in Henan Province, China. The survey was conducted in two stages. The first stage was the pre-investigation stage. In April 2021, 30 family farms in Henan Province were randomly selected for household interviews to pre-test the questionnaire to delete unclear questions and add additional questions. A mixed team of five enumerators, majoring in agricultural economics or ecological psychology, was chosen to collect the data. To improve data accuracy, all the enumerators attended a three-day training workshop. The clarity of the questionnaire was improved after this stage. The second stage was the formal survey, which was conducted from May to October 2021. A stratified random sampling method was used to gather data. First, all the selected counties in Henan province were sorted according to regional GDP and divided into five categories: very high, relatively high, medium, relatively low, and very low. Four counties were randomly selected from each category (as shown in Figure 2). Then, two townships were randomly selected from each sampled county. Next, four sample villages were randomly selected from each sampled township. Finally, 10 family farms were randomly selected from each sampled village. Therefore, the sample for Henan province covered 20 counties, 40 townships, 80 villages, and 800 family farms. Overall, 800 questionnaires were distributed, and 653 valid questionnaires were returned. A validity rate of 81.6% was achieved after eliminating questionnaires that omitted key information or presented self-contradictory information (for instance, where age is less than the number of years of education).

In this paper, the data were collected through face-to-face interviews to reduce the response bias and improve the response rate. In the process of the interview, first, interviewers asked vegetable farmers whether they use GCTs, whether they have heard of GCTs for the vegetable plant, under what circumstances they are willing to adopt the technology and whether there are subsidies and training. For interviewees who answered seriously without contradictory words, we would allow them to fill in the questionnaire. Each interviewee would have about 20 min to fill in it. For those who are illiterate, interviewers completed the interview by asking for items in the questionnaire. In the process of inquiry, our interviewers did not convey any guiding information, to ensure the results are objective and fair. Second, interviewees were assured that they had the right to refuse the interview and told that interviewers were researchers rather than officials. In this way, the psychological burden of the interviewees was reduced. Additionally, they were told that the content of the questionnaire was closely related to their lives and the results of the interview were very useful for policy-making; as such, the interviewees would have a positive impression of our survey (Lou et al., 2021). This helped to improve the accuracy of their response. Moreover, we emphasized that the interview was completely anonymous and the results were for research purposes rather than commercial use so that the respondents knew that their privacy was well protected. After the interview, our interviewers would sort out and analyze the results, and delete those questionnaires with a high repetition rate and with many missing values. For controversial questionnaires, our three researchers discussed and determined whether the questionnaire should be left.

### 3.3. Sample description

As shown in Table 1, from the perspective of individual basic characteristics, in terms of gender, males accounted for 74.7% and females accounted for 25.3%. In terms of age, farmers aged 40–50 accounted for the highest proportion (67.69%), with an average age of about 46.04. In terms of education level, farmers’ average education years is about 10.7, and the education level is generally low. The main characteristics of the samples and their distribution are shown in Table 1. In addition, the number of farming households with more than 2 members accounted for 58.65% of the total sample, indicating that the number of farming households with more than 2 members was relatively small. The number of farming households joining cooperatives accounted for 47.47% of the sample, indicating that the current farming households are not highly organized. The number of farming households that often participate in agricultural orders only accounts for 2.76% of the total sample, and the degree of farming households’ participation in agricultural orders is generally low. Furthermore, the statistical results showed that 88.82% of the farmers adopted green prevention and control technologies (GCTs). Among them, 34.61% of the farmers occasionally and frequently adopted GCTs, 22.97% of the farmers often adopted it, and only 31.24% of the farmers always adopted it (the sum of the three = 88.82), which shows that the proportion of farmers adopting GCTs in the sample area is high, but the degree of adoption is not high. Then, the motives were measured by exploratory factor analysis using SPSS 24.0 software. The measured KMO value was 0.676, and the Bartlett sphericity test passed the 1% significance test, indicating that factor analysis could be conducted. Two common factors with characteristic roots greater than one were also obtained. The variance contribution rate of the common factor 1 was 43.796%, which focused on the adoption of GCTs is conducive to improving vegetable quality, obtaining green certification, increasing planting income and saving production costs, i.e., farmers’ “economic efficiency motivation.” The contribution rate of variance of common factor 2 is 26.622%, which focuses on the local penalties for agricultural surface pollution and government subsidies for green and efficient technology promotion services, i.e., farmers’ “environmental regulatory arbitrage motivation.” The composite index value was obtained: Motivation = (economic efficiency motivation x 43.796% + regulatory arbitrage motivation x 26.622%)/70.418%.

**Table 1:** Variable definitions and descriptive statistics.

Variable type	Variable		Measure	Mean	Std. dev.
**Dependent variable**	GCTs adoption behavior		1 = Never, 2 = Occasionally, 3 = Frequently, 4 = Often, 5 = Always	3.485	1.356
**Main independent variable**	Information acquisition	Access to information: government, cooperatives, neighborhoods, media	0 = None, 1 = 1 information channel, 2 = 2 information channels, 3 = 3 information channels, 4 = 4 information channels	2.613	1.366
	Motivation	Conducive to improving the quality of vegetables	1 = yes; 0 = no	0.557	0.497
		Conducive to obtaining green certification	1 = yes; 0 = no	0.620	0.486
		Conducive to increasing planting income	1 = yes; 0 = no	0.606	0.489
		Conducive to save production cost	1 = yes; 0 = no	0.515	0.500
		The government subsidizes green technology promotion services	1 = yes; 0 = no	0.596	0.491
		There are local penalties for agricultural non-point source pollution	1 = yes; 0 = no	0.455	0.498
	Behavioral skills	Participate in various agricultural cultivated land protection technology training	1 = yes; 0 = no	0.470	0.499
		Often communicate with agricultural technicians	1 = yes; 0 = no	0.455	0.498
		Often study publicity materials of green prevention and control technology	1 = yes; 0 = no	0.459	0.499
**Control variable**		Gender	1 = male; 0 = female	0.747	0.435
		Age	Age in 2021	46.044	5.481
		Educational level	Years of education	10.698	2.819
		Number of laborers	Total number of family vegetable planting laborers	2.538	0.667
		The main relatives are party members or village cadres	1 = yes; 0 = no	0.158	0.365
		The local government has incentives for green planting	1 = yes; 0 = no	0.492	0.500
		Degree of participation in agricultural orders	1 = Never, 2 = Occasionally, 3 = Frequently, 4 = Often, 5 = Always	1.995	0.901

### 3.4. Model construction

#### 3.4.1. Baseline model

The explanatory variables in this paper belong to ordered multi-classification variables. Referring to Shi and Zhang (2022)’s method of dealing with the problem that the explained variables are ordered multi-classification variables, this paper uses the ordered logistic regression model to analyze the adoption behavior of farmers’ GCTs. The specific expressions are as follows.


(1) Logit(Ρi)=Ln[Ρ(y≤i)/Ρ≥(Υj+i)]=αi+βx

where Pj=P(y=j); X is the variable affecting the adoption behavior of green control technology GCTs by farmers; β is the regression coefficient corresponding to X; and $\alpha_{i}$ is the intercept of the model. After obtaining the parameter estimates, the probability of occurrence of Υ=ican be obtained by the following equation.


(2) $$Ρ\left(y\leq j/x\right)=\frac{e^{-\left(\alpha +\beta xi\right)}}{1+e^{-\left(\alpha +\beta xi\right)}}$$

#### 3.4.2. Mediating effect test

The mediating effect refers to the fact that the relationship between variables is not a direct causal chain but can be influenced indirectly through other variables. Compared with the direct influence relationship between variables, the mediating effect model can analyze the influence process and the mechanism of variables more deeply. Given that the mediating variables “motivation” and “behavioral skills” are continuous variables, the transmission mechanism of motivation and behavioral skills in influencing farmers’ adoption of GCTs is tested by the stepwise regression method according to Wen and Ye (2014). The model was constructed as follows.


(3) Υ=cΧ+e1

(4) Μ=aΧ+e2

(5) Υ=c'Χ+bΜ+e3

where Χ is the independent variable, Μis the mediating variable, and Y is the dependent variable. $e_{1},e_{2},e_{3}$ are the regression residuals.c is the total effect of the independent variable on the dependent variable; a is the effect of the independent variable on the mediating variable; b is the effect of the mediating variable on the dependent variable; and $c^{\prime }$ is the direct effect of the independent variable on the dependent variable.

## 4. Results and analysis

### 4.1. Baseline model regression

#### 4.1.1. Analysis of model results

Stata15.0 was used to analyze the data in the paper. The model calculation results show (Table 2) that the behavior logic of farmers’ GCTs adoption behavior follows the IMB model, and the significance of the log-likelihood ratio test of the model was found to be less than 0.05, indicating that the overall fit of the model was good.

**Table 2:** Estimation of model results.

Variable type	Model 1	Mode 2	Mode 3
Information acquisition	0.917^***^(0.064)		
Motivation		0.808^***^(0.106)	
Behavioral skills			0.877^***^(0.081)
Gender	0.174 (0.172)	0.082 (0.169)	0.112 (0.168)
Age	0.005 (0.014)	0.012 (0.013)	−0.00009 (0.013)
Educational level	0.008 (0.026)	0.021 (0.026)	0.037 (0.026)
Number of laborers	0.022 (0.111)	0.043 (0.107)	−0.013 (0.107)
Degree of participation in agricultural orders	0.243^***^(0.088)	0.434^***^(0.086)	0.469^***^(0.087)
The main relatives are party members or village cadres	0.242 (0.205)	0.155^*^(0.204)	0.207 (0.202)
The local government has incentives for green planting	0.509^***^(0.148)	0.541^***^(0.144)	0.667^***^(0.145)
Log-likelihood	−876.245	−964.528	−930.046
Pseudo R2	0.134	0.046	0.081
Prob>chi2	0.000	0.000	0.000

***p < 0.01; **p < 0.05; *p < 0.1, standard errors in parentheses.

As seen in Table 2, Model 1 shows that the information acquisition channels of farmers have a direct driving effect on the GCTs adoption behavioral response (β = 0.917, *p* < 0.000), indicating that the wider the source of information is, the greater the range of effective information that farmers are exposed to in the market, and with the transfer of information, farmers will tend to compare and imitate the behavior of others, resulting in a convergence of GCTs adoption behavior among farmers (hypotheses H1was supported). Model 2 shows that motivation has a direct driving effect on farmers’ GCTs adoption behavior (β = 0.808, *p* < 0.000), indicating that maximizing economic benefits and minimizing risks are the fundamental motivations for farmers to produce, and farmers will tend to adopt GCTs to avoid losses (hypotheses H2 was supported). Model 3 shows that the behavioral skills of farmers have a direct driving effect on the GCTs adoption behavioral response (β = 0.877, *p* < 0.000), indicating that mastering GCTs can effectively improve farmers’ adoption behavior (hypotheses H3 was supported).

Furthermore, in Models 1–3, the control variables “participation in agricultural orders, government incentives for green farming and the main relatives are party members or village cadres” passed the significance test. First, participating in agricultural orders can reduce production risks and additional costs, which meet the production requirements of large-order farmers, so farmers who have participated in order or standardized production will be more inclined to adopt GCTs in agriculture. In addition, based on economic incentives, constraints and punishments, government incentives with the support of production technology, insurance, service and sales have a significant role in promoting GCTs adoption behavior. Moreover, from the perspective of social relations, if farmers have close relations (relatives, good friends, partners, etc.) with village (town) cadres and rural talents, these relations may help farmers understand and accept GCTs information, and then affect farmers’ willingness to adopt GCTs.

#### 4.1.2. Robustness test

To ensure the robustness of the results, the empirical results are tested in the paper. First, the robustness of the model is tested by replacing the model with the ordered Probit model. Second, the latent variables in each core variable are changed for testing. For example, the variable “access to information: government, cooperative, neighborhood” is replaced with “access to information: government, cooperative, neighborhood.” In the motivation, the core latent variables of “no additional cost for green control technology adoption” and “local penalties for agricultural surface pollution,” which represent the motivation of economic benefits and environmental regulatory arbitrage motivation, were removed. In the behavioral technique, the core latent variable of “participation in various types of training such as agricultural farmland protection technology” was removed. As shown in Table 3, the results obtained by the two methods are basically consistent with the results in Table 2, indicating that the estimation results are robust.

**Table 3:** Robustness test results.

Variable type	Ordered probit test			Variable replacement test		
	Model 4	Model 5	Model 6	Model 7	Model 8	Model 9
Information acquisition	0.535^***^(0.035)			1.292^***^(0.090)		
Motivation		0.473^***^(0.061)			0.710^***^(0.080)	
Behavioral skills			0.510^***^(0.046)			0.738^***^(0.077)
Log likelihood	−870.924	−963.646	−930.560	−869.818	−952.755	−945.495
Pseudo R2	0.139	0.047	0.080	0.140	0.058	0.065
Prob>chi2	0.000	0.000	0.000	0.000	0.000	0.000

***p < 0.01; **p < 0.05; *p < 0.1, standard errors in parentheses.

### 4.2. Results of the mediating effect test

The results of mediating effect test are reported in Table 4.

**Table 4:** Mediating effect estimation results.

**Variable type**	GCTs adoption behavior	Motivation	GCTs adoption behavior	Behavioral skills	GCTs adoption behavior	GCTs adoption behavior	Behavioral skills	GCTs adoption behavior
**Information acquisition**	0.569^***^	0.134^***^	0.528^***^	0.222^***^	0.476^***^			
	0.032	0.020	0.032	0.028	0.031			
**Motivation**			0.307^***^			0.555^***^	0.444^***^	0.314^***^
			0.060			0.069	0.052	0.067
**Behavioral skills**					0.420^***^			0.542^***^
					0.042			0.049
**Control variables**	Controlled			Controlled		Regression result		

***p < 0.01; **p < 0.05; *p < 0.1.

(1) In the path of “information-motivation-farmers’ GCTs adoption behavior,” the coefficient value of information on motivation is 0.134 (*p* < 0.000), suggesting that information acquisition has a significant positive effect on farmers’ GCTs adoption motivation (Hypothesis H4 is further verified). In addition, the coefficient value of information on farmers’ GCTs behavior is 0.569 (*p* < 0.000), and information still had a significant positive effect on farmers’ GCTs (*β* = 0.528, *p* < 0.000) after the inclusion of motivation variables. Thus, it is seen that the mediating effect of motivation on the relationship between information and farmers’ GCTs is significant, and the mediating effect is 0.041 (hypothesis H5 is valid).

(2) In the path of “information-behavioral skills-farmers’ GCTs adoption behavior,” the coefficient value of information on behavioral skills is 0.222 (*p* < 0.000), suggesting that information acquisition has a significant positive effect on farmers’ GCTs behavioral skills (Hypothesis H6 is further verified). In addition, information still had a significant positive effect on farmers’ GCTs adoption behavior (*β* = 0.476, *p* < 0.000) after the inclusion of behavioral skills variables, indicating that information acquisition can act on farmers’ GCTs adoption behavior through behavioral skills mediation, and the mediating effect was 0.093 (hypothesis H7 is valid).

(3) In the path of “motivation-behavioral skill farmers’ GCTs adoption behavior,” the coefficient value of motivation on behavioral skill is 0.444 (*p* < 0.000), suggesting that motivation has a significant positive effect on farmers’ GCTs behavioral skills (Hypothesis H8 is further verified). In addition, motivation positively influences farmers’ GCTs adoption behavior (*β* = 0.555, *p* < 0.000), and motivation still has a significant positive influence on farmers’ GCTs adoption behavior (*β* = 0.314, *p* < 0.000) after the inclusion of behavioral skill variables, indicating that motivation can act on farmers’ GCTs adoption behavior through behavioral skills mediation, and the mediating effect is 0.241 (hypothesis H9 is valid).

Then, the robustness test of the mediating effect was conducted using bootstrap (n = 5,000). The standard of the test is to see whether the confidence interval contains 0. If it does, the original hypothesis should be rejected. As seen in Table 5, the results obtained were generally consistent.

**Table 5:** Results of bootstrap robustness test.

Influence path	Effect	Regression coefficient	SE	95% CI	
				LLCI	ULCI
Information Acquisition→Motivation→Farmers’ GCTs Adoption Behavior	Total effect	0.569	0.032	0.507	0.631
	Direct effect	0.528	0.032	0.465	0.591
	Indirect effect	0.041	0.011	0.022	0.064
Information Acquisition→Behavioral Skills→Farmers’ GCTs Adoption Behavior	Total effect	0.569	0.032	0.507	0.631
	Direct effect	0.476	0.031	0.415	0.536
	Indirect effect	0.093	0.015	0.066	0.125
Motivation→Behavioral Skills→Farmers’ GCTs Adoption Behavior	Total effect	0.555	0.069	0.419	0.690
	Direct effect	0.314	0.067	0.183	0.445
	Indirect effect	0.241	0.034	0.177	0.308

## 5. Conclusion and discussion

### 5.1. Conclusion

Comprehensive promotion of agricultural GCTs is an important measure to ensure the safety of the agricultural ecological environment and achieve high-quality agricultural development. However, due to the unknown risks and information asymmetry of new technologies, the actual degree of adoption by “rational” farmers is not high. To further promote the comprehensive promotion of GCTs in agricultural production, the search for effective ways to improve farmers’ adoption behavior of GCTs has become the core issue of the current research. Unlike the direct linear analysis of farmers’ GCTs adoption behavior in the previous literature, this paper takes behavioral intervention as the key to determining the behavior change from the root of the problem and analyzes farmers’ GCTs adoption behavior based on the “information-motivation-behavior skill” (IMB) intervention model (Fisher and Fisher, 1992, 2000; Fisher et al., 2006, 2014) and draws the following main conclusions.

First, a total of 88.82% of farmers in the sample area actively adopted GCTs, but the adoption level of GCTs was low, with only 30.7% of farmers regularly adopting GCTs. Second, information, motivation, and behavioral skills have significant positive effects on farmers’ adoption behavior of GCTs. It was found that farmers with wide access to information (*β* = 0.917, *p* < 0.000), strong behavioral motivation (*β* = 0.808, *p* < 0.000), and better mastery of agricultural technology (*β* = 0.877, *p* < 0.000) were more likely to adopt GCTs, which is consistent with the prediction of the classical IMB model (Fisher and Fisher, 1992, 2000; Fisher et al., 2006, 2014; Seacat and Northrup, 2010; Ehret et al., 2021). Furthermore, motivation and behavioral skills are activated through information, and the more information access channels there are, the greater the activation and the stronger the facilitation effect on farmers’ green control technology adoption behavior, which is consistent with the findings of Shi and Zhang (2022) and Zhang et al. (2020). In addition, motivation also has a facilitating effect on behavioral skills (*β* = 0.444, *p* < 0.000), which can indirectly affect farmers’ GCTs adoption behavior through behavioral skills. In summary, this study confirms that the theoretical framework of the information–motivation–behavioral skills (IMB) model is applicable to the study of vegetable farmers’ adoption behavior of green prevention and control techniques (GCTs); specifically, that the adoption of the behavioral intention of vegetable farmers is affected by GCTs information acquisition, adoption motivation and behavior skills. This research broadens the research scope of IMB as well as sustainable technologies such as green prevention and control techniques (GCTs). It also draws more attention to the academic study of vegetables and will hopefully motivate later generations to further study the behavior of vegetable farmers.

### 5.2. Theoretical contributions

Our study makes several contributions to the literature. First, in response to some studies (Lou et al., 2021; Yu et al., 2021a; Niu et al., 2022) that called for future research examining factors influencing farmers’ intention to adopt pro-green control technology using various theoretical frameworks and diverse populations, our study is the first to use the information–motivation–behavioral skills (IMB) model as the theoretical framework for exploring factors influencing vegetable farmers’ GCTs adoption behavior. Secondly, the findings are consistent with the IMB model (Seacat and Northrup, 2010; Ehret et al., 2021), supporting the model’s utility in explaining green prevention and control techniques behavior. More specifically, our study contributes to the literature by validating the utility of the IMB model in predicting and explaining vegetable farmers’ GCTs adoption behavior in the context of China, where the use of GCTs remains mainly experimental and implemented at a small scale, and providing a theoretical ground for future research. Finally, previous studies offered a holistic understanding of pro-green control technology adoption behavior by exploring farmers’ own resource endowment (Xiong and He, 2020), social networks (Gao et al., 2019a), economic incentives (Geng et al., 2017) and technical characteristics (Gao et al., 2020; Liu et al., 2022) in general. By focusing on psychologically-based interventions, our study contributes to the existing body of literature in the field of green production. Among the three IMB predictors, information acquisition had the most dominant and direct effect on pro-green control technology adoption behavior. It is also worth noting that both direct and indirect effects were statistically significant. Our results support the practicability of applying the IMB model to predict green prevention and control techniques adoption intentions, indicating that the IMB model can be used as a framework for developing educational interventions promoting pro-green control technology by farmers.

### 5.3. Policy recommendations

This study can help decision-makers, such as people in the Chinese government, better understand the factors affecting vegetable farmers’ adoption behavior of green prevention and control techniques (GCTs) and make targeted GCTs extension policies. First, the policymakers should strengthen the construction of rural information resource-sharing projects, improve information infrastructure, and meet the information needs of farmers as much as possible. In addition, carry out special education and training on information literacy that meets the characteristics of farmers to effectively improve their information literacy so that rich, timely and accurate information can help farmers reduce costs and increase income to the greatest extent. Second, strengthen the publicity and promotion of GCTs, mobilize farmers to participate in the enthusiasm, and take the initiative to learn and master green prevention and control technology. At the same time, agricultural technology subsidies should be increased, the perceived risk level of farmers’ adoption of GCTs should be weakened, and farmers should be guided to correctly understand the effect of yield increase brought by green prevention and control technology. Third, the reform of the agricultural extension system should be deepened, technical guidance and training in green prevention and control for farmers should be strengthened, farmers’ scientific knowledge of GCTs should be improved, and the adoption rate of GCTs should be increased among farmers. Moreover, although policy support or technology information service is important, the effect of policy or technology implementation needs more attention. We need to form a vertical information feedback mechanism, effectively understand the real needs and difficulties of farmers in technology and policies, and provide farmers with full-course or phased services. Environmental pollution caused by pesticide abuse in the agricultural sector has also become a major problem facing countries worldwide. Our study is relevant in these times, and our results suggest the need for a transition to green prevention and control techniques (GCTs), the reduction of pesticide use, and the implementation of government programs. It is crucial for the government to strongly support and promote farmers’ awareness to encourage the transition to green production. This paper suggests strategies for the Chinese government to potentially influence vegetable farmers’ behavior; however, we believe that green production technology has no international boundaries and that other governments can also take corresponding measures to strengthen the promotion and application of GCTs.

### 5.4. Research limitations and outlook

Our study aims to solve the problem of environmental pollution and pest invasion and provide strategies for government departments to promote the adoption of green prevention and control techniques. By doing these, we strive to achieve the goal of full coverage of GCTs and sustainable development in the agricultural section worldwide in the future. GCTs is the localized version of IPM in China. Similar to China, other developing countries are also facing the challenges of chemical pesticide overuse and low levels of IPM adoption. Thus, this study’s analytical framework may be applicable to other developing countries, and the conclusions may have important implications for these countries as they implement IPM promotion policies. Of course, we have to acknowledge that this study has some limitations that need to be improved upon in future research work. First, the findings of this paper are based on survey data of vegetable farmers in Henan Province, but it remains to be seen whether consistent findings can be drawn in other regions of China. Second, GCTs is a complex technology package that includes multiple seed technologies. This paper only examines whether farmers adopt GCTs, and further research could include specific GCTs sub-technologies in the analysis framework to explore the contribution of information accessibility to farmers’ GCTs adoption behavior. Third, it is important to note that the article treats farmers as the main body of adoption of agricultural green prevention and control technologies, but rural environmental management and agricultural green prevention and control technology promotion cannot be solved by farmers alone but also requires coordination among various sectors, such as government, the market, and the rural communities. Further research and improvement are needed on how each sector can “play” their respective roles to give full play to the “information-motivation-behavior skills” to promote farmers’ adoption of green control technologies. Furthermore, to best strengthen IMB interventions, future research will also need to consider additional contextual and individual-level factors that may moderate different IMB components, such as farmers’ value demands, attention allocation, risk aversion and business scale.

## Data availability statement

The original contributions presented in the study are included in the article/supplementary material, further inquiries can be directed to the corresponding author.

## Ethics statement

Ethical review and approval were not required for the study on human participants in accordance with the local legislation and institutional requirements. The participants provided their written informed consent to participate in this study.

## Author contributions

TC and XL: conceptualization, data curation, and writing-review and editing. XL and ZW: methodology. TC: formal analysis and writing–original draft preparation. TC, ZW, and JZ: investigation. ZW: supervision. All authors have read and agreed to the published version of the manuscript.

## Funding

This work was supported by the General Project of Humanities and Social Sciences Research in Universities of Henan Province under Grant 2023-ZZJH-027, the Key Project of Educational Science Planning in Henan Province under Grant 2023JKZD41, the Research Project of Henan Social Science Association under Grant SKL-2022-2774, the Teacher Development Research and Practice Project of Henan Polytechnic Institute under Grant JSFZ202202, the Vocational Education Research Project of Henan Polytechnic Institute under Grant 2022ZJYJ12, and the Science and Technology Project of Science and Technology Department of Henan Province (Research on the Mechanism and Path of Green Finance Helping Henan Ecological Product Value Realization under the Goal of “Double Carbon”). The funders had no role in the study design, data collection and analysis, decision to publish, or preparation of the manuscript.

## Conflict of interest

The author declares that the research was conducted in the absence of any commercial or financial relationships that could be construed as a potential conflict of interest.

## Publisher’s note

All claims expressed in this article are solely those of the authors and do not necessarily represent those of their affiliated organizations, or those of the publisher, the editors and the reviewers. Any product that may be evaluated in this article, or claim that may be made by its manufacturer, is not guaranteed or endorsed by the publisher.
